# Fibroblast and keratinocyte gene expression following exposure to the extracts of holy basil plant (*Ocimum tenuiflorum*), malabar nut plant (*Justicia adhatoda*), and emblic myrobalan plant (*Phyllanthus emblica*)

**DOI:** 10.1016/j.dib.2017.12.055

**Published:** 2018-01-04

**Authors:** Takao Someya, Katsura Sano, Kotaro Hara, Yoshimasa Sagane, Toshihiro Watanabe, R.G.S. Wijesekara

**Affiliations:** a ALBION Co. Ltd., 1-7-10 Ginza, Chuo-ku, Tokyo 104-0061, Japan; b Department of food and Cosmetic Science, Faculty of Bioindustry, Tokyo University of Agriculture, 196 Yasaka, Abashiri, Hokkaido 099-2493, Japan; c Department of Aquaculture and Fisheries, Faculty of Livestock, Fisheries and Nutrition, Wayamba University of Sri Lanka, Makandura, Gonawila 60170, Sri Lanka

**Keywords:** Real-time PCR, Gene expression profile, Fibroblast, Keratinocyte, Holy basil extract, *Ocimum tenuiflorum*, Maduruthala, Malabar nut plant extract, *Justicia adhatoda*, Adayhoda, Emblic myrobalan extract, *Phyllanthus emblica*, Nelli

## Abstract

This data article provides gene expression profiles, determined by using real-time PCR, of fibroblasts and keratinocytes treated with 0.01% and 0.001% extracts of holy basil plant (*Ocimum tenuiflorum*), sri lankan local name “maduruthala”, 0.1% and 0.01% extracts of malabar nut plant (*Justicia adhatoda*), sri lankan local name “adayhoda” and 0.003% and 0.001% extracts of emblic myrobalan plant (*Phyllanthus emblica*), sri lankan local name “nelli”, harvested in Sri Lanka. For fibroblasts, the dataset includes expression profiles for genes encoding hyaluronan synthase 1 (HAS1), hyaluronan synthase 2 (HAS2), hyaluronidase-1 (HYAL1), hyaluronidase-2 (HYAL2), versican, aggrecan, CD44, collagen, type I, alpha 1 (COL1A1), collagen, type III, alpha 1 (COL3A1), collagen, type VII, alpha 1 (COL7A1), matrix metalloproteinase 1 (MMP1), acid ceramidase, basic fibroblast growth factor (bFGF), fibroblast growth factor-7 (FGF7), vascular endothelial growth factor (VEGF), interleukin-1 alpha (IL-1α), cyclooxygenase-2 (cox2), transforming growth factor beta (TGF-β), and aquaporin 3 (AQP3). For keratinocytes, the expression profiles are for genes encoding HAS1, HAS2, HYAL1, HYAL2, versican, CD44, IL-1α, cox2, TGF-β, AQP3, Laminin5, collagen, type XVII, alpha 1 (COL17A1), integrin alpha-6 (ITGA6), ceramide synthase 3 (CERS3), elongation of very long chain fatty acids protein 1 (ELOVL1), elongation of very long chain fatty acids protein 4 (ELOVL4), filaggrin (FLG), transglutaminase 1 (TGM1), and keratin 1 (KRT1). The expression profiles are provided as bar graphs.

**Specifications Table**

**Table:** 

Subject area	*Biology*
More specific subject area	*Cell biology*
Type of data	*Graph*
How data was acquired	*Quantitative RT-PCR (LightCycler 96 system, Roche)*
Data format	*Analyzed*
Experimental factors	*Isolation of total cellular RNA, cDNA amplification, PCR analysis*
Experimental features	*Analysis of gene expression by quantitative RT-PCR*
Data source location	*Negombo, Sri Lanka*
Data accessibility	*Data are available within this article*

**Value of the data**

• Data showing changes in gene expression levels in response to holy basil (*Ocimum tenuiflorum*) extract, malabar nut (*Justicia adhatoda*) extract and emblic myrobalan (*Phyllanthus emblica*) extract exposure are valuable for estimating effects of the extract on fibroblasts and keratinocytes.
• The data presented in this article showing that holy basil (*Ocimum tenuiflorum*) extract, malabar nut (*Justicia adhatoda*) extract and emblic myrobalan (*Phyllanthus emblica*) extract up- or down-regulates the expression of genes involved in epidermal and dermal cells could be important for investigations in pharmacology and cosmetics.
• The present data can be referenced by investigations into chemicals and natural medicines for the epidermal and dermal tissues.
• The data in this article provides useful knowledge for the cosmeceutical application of holy basil extract, malabar nut extract and emblic myrobalan, traditional ayurvedic plants in Sri lanka.

## 1 Data

**Fig. 1:**
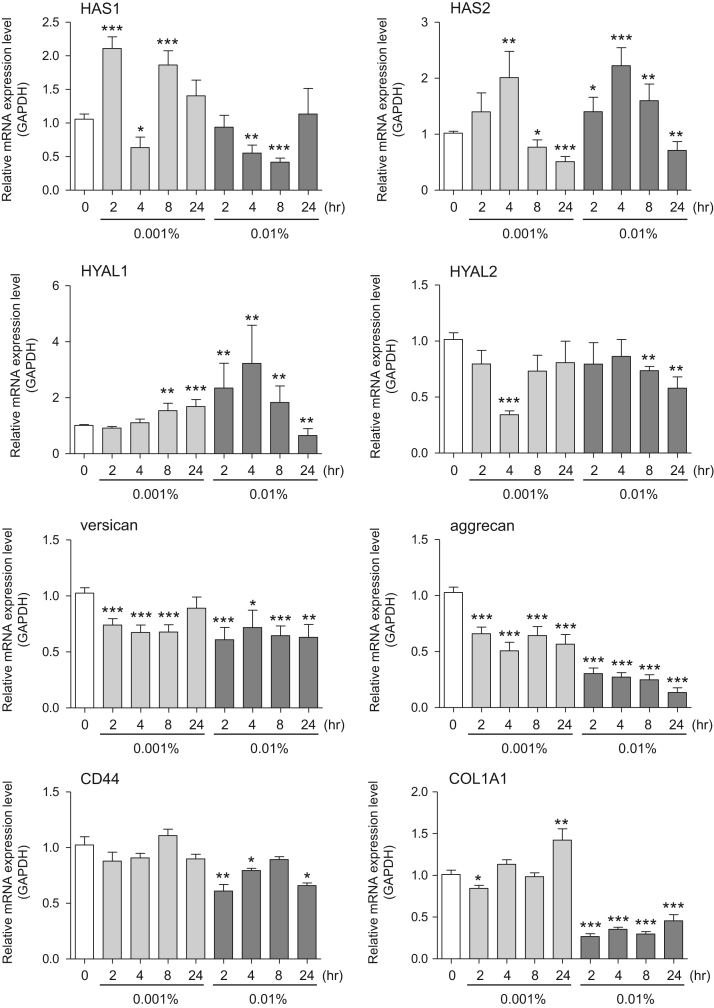

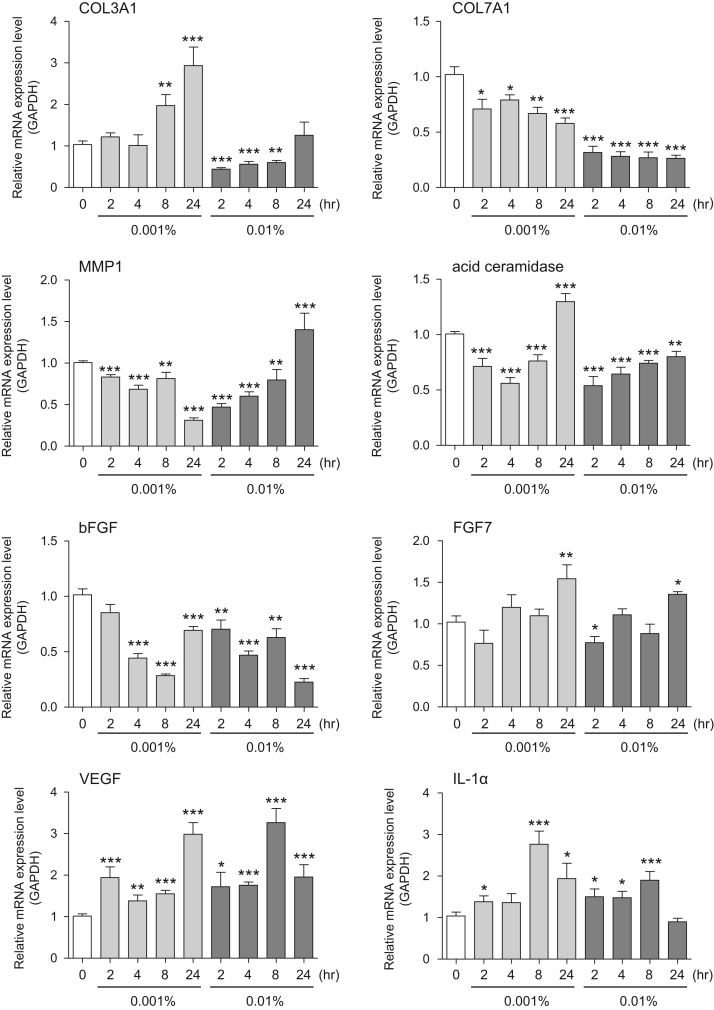

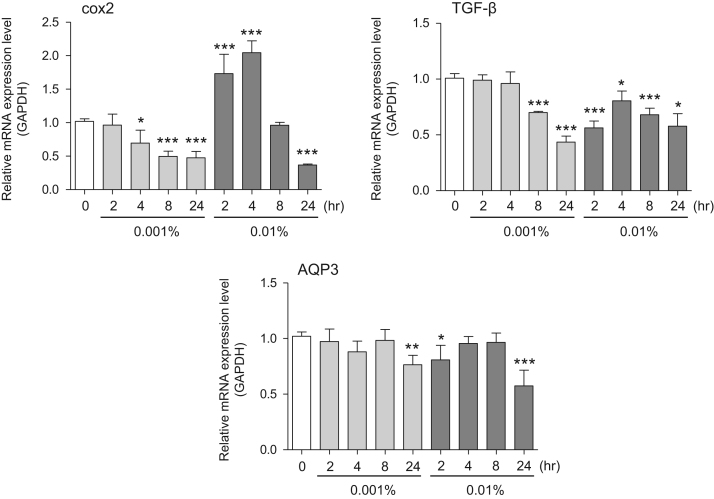
Gene expression levels in fibroblast cells after exposure to holy basil extract. The mRNA expression levels were normalized to GAPDH expression, and the relative gene expression levels in the cells at 2, 4, 8, and 24 h after initiation of extract exposure were compared to the corresponding levels for unexposed cells, whose levels were defined as 1.0.

**Fig. 2:**
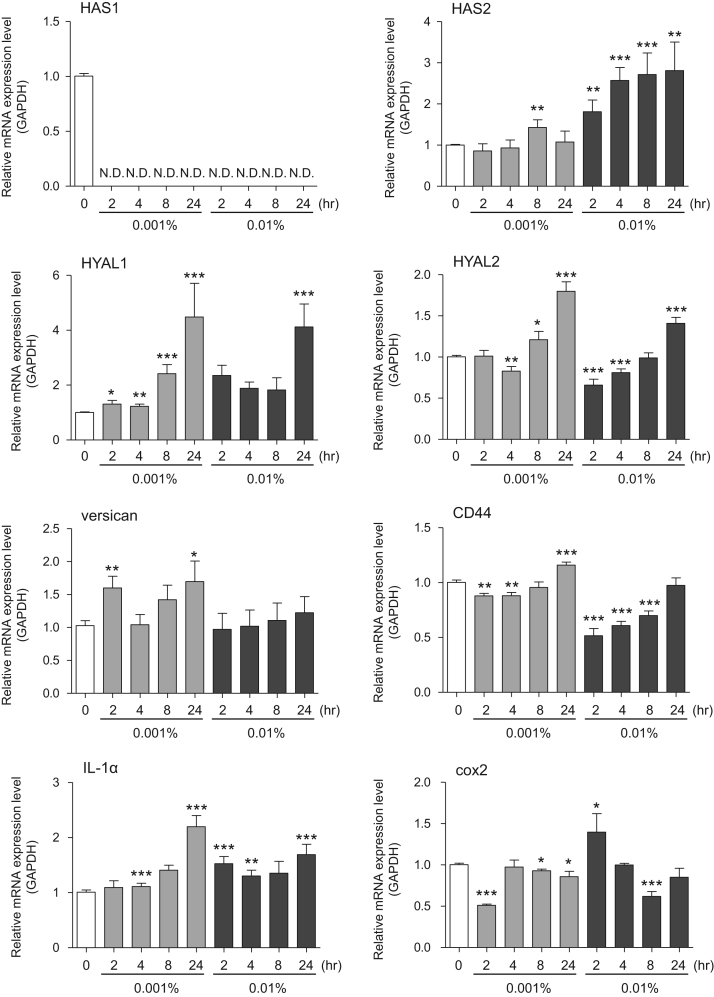

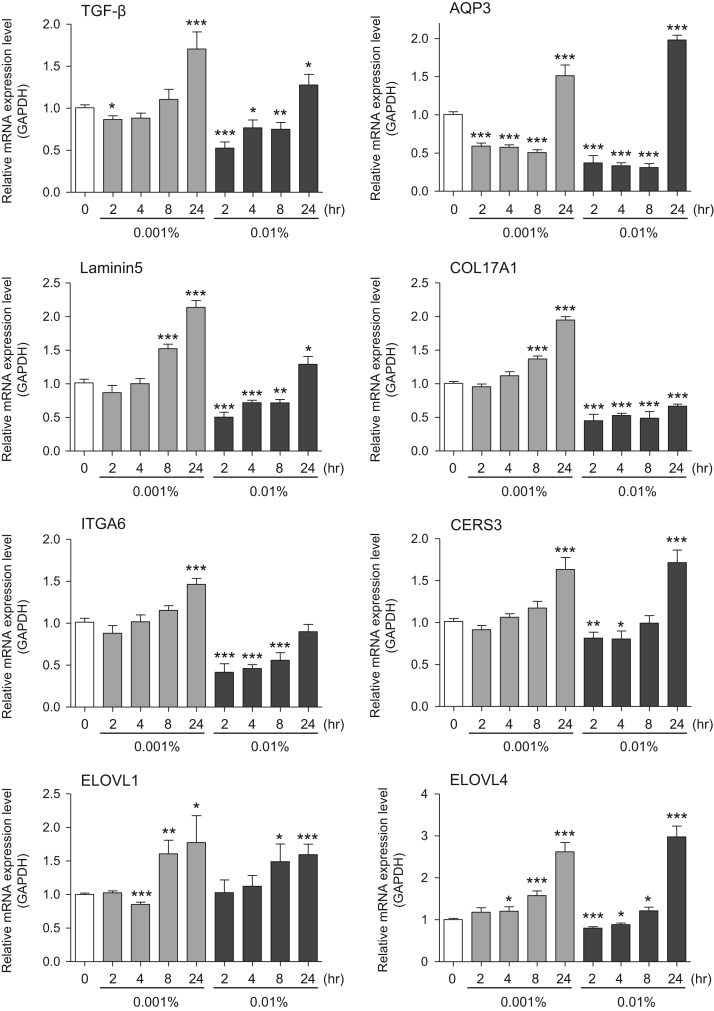

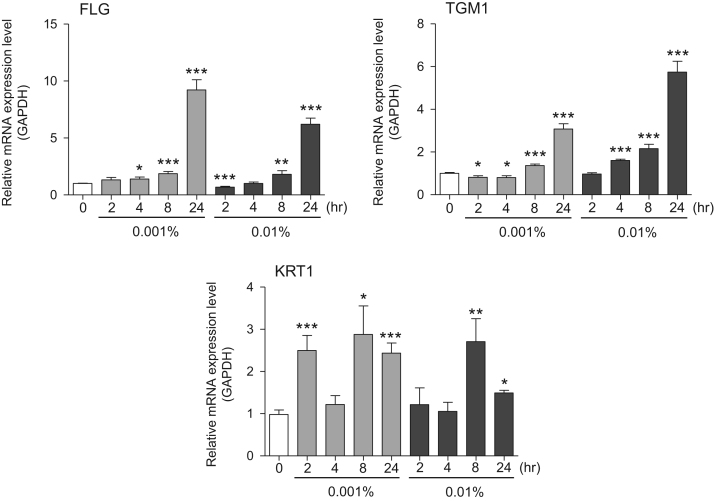
Gene expression levels in keratinocyte cells after exposure to holy basil extract. The mRNA expression levels were normalized to GAPDH expression, and the relative gene expression levels in the cells at 2, 4, 8, and 24 h after initiation of extract exposure were compared to the corresponding levels for unexposed cells, whose levels were defined as 1.0. N.D. = not detected.

**Fig. 3:**
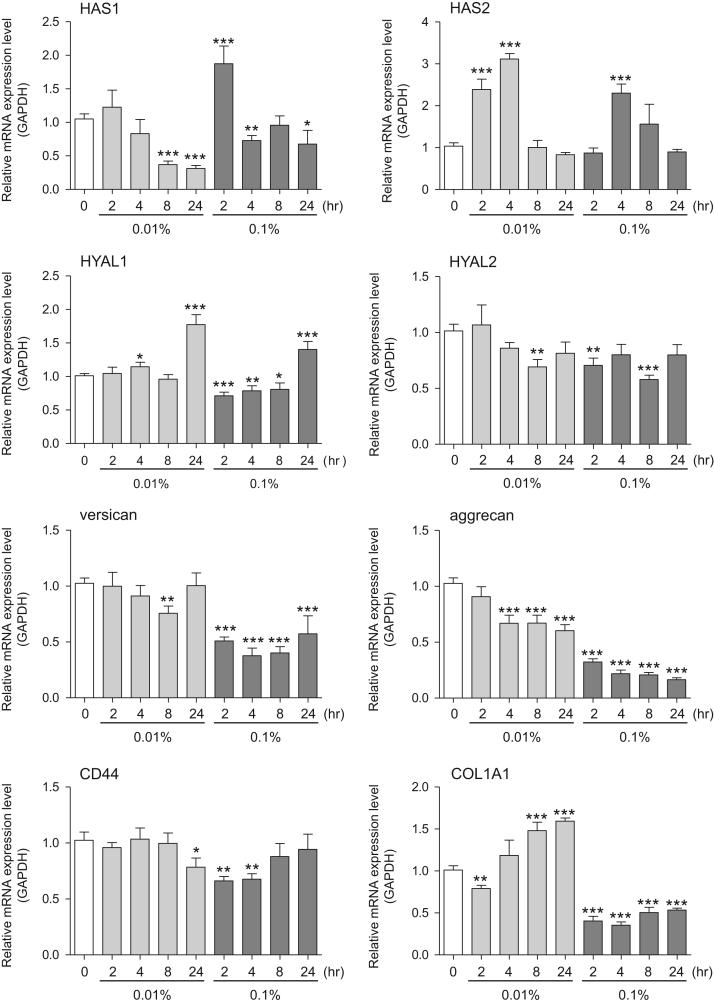

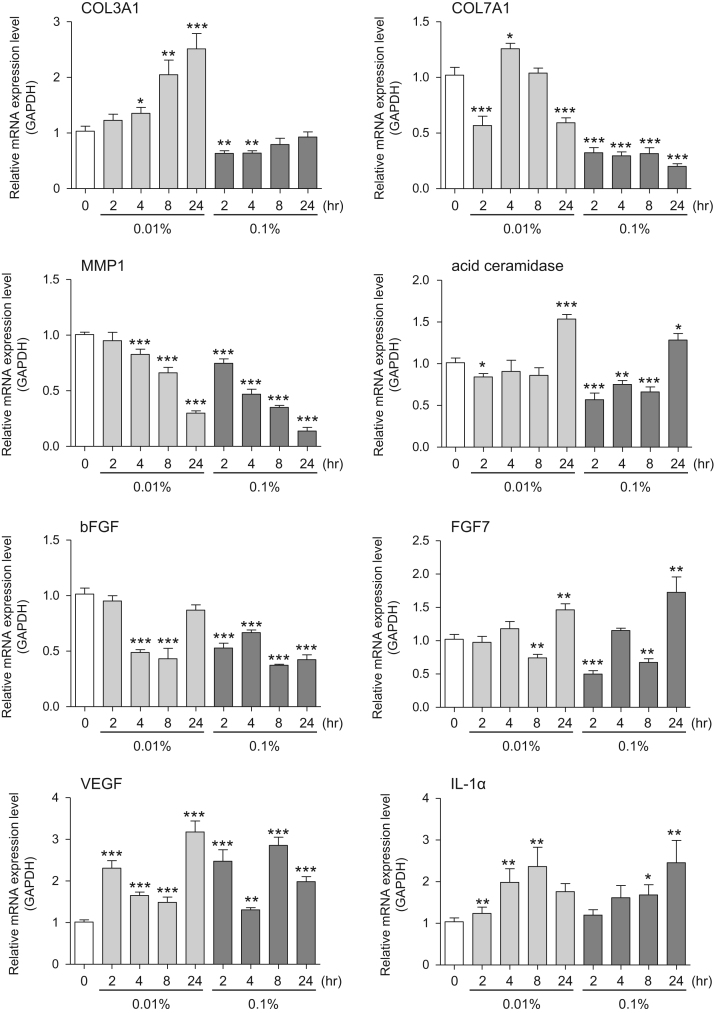

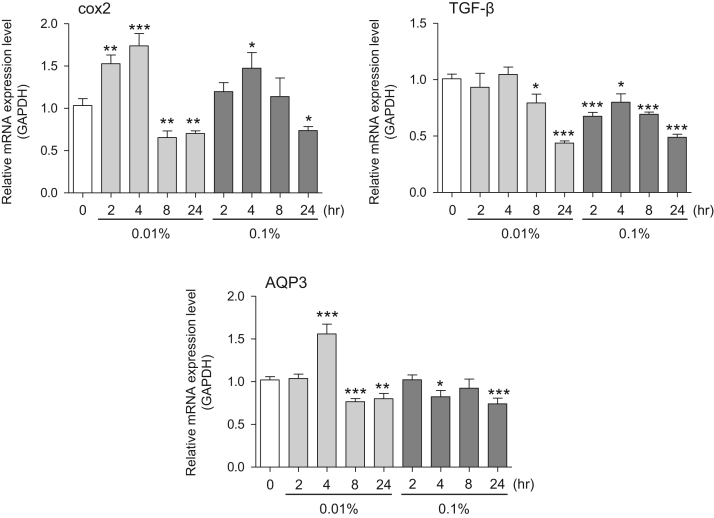
Gene expression levels in fibroblast cells after exposure to malabar nut extract. The mRNA expression levels were normalized to GAPDH expression, and the relative gene expression levels in the cells at 2, 4, 8, and 24 h after initiation of extract exposure were compared to the corresponding levels for unexposed cells, whose levels were defined as 1.0.

**Fig. 4:**
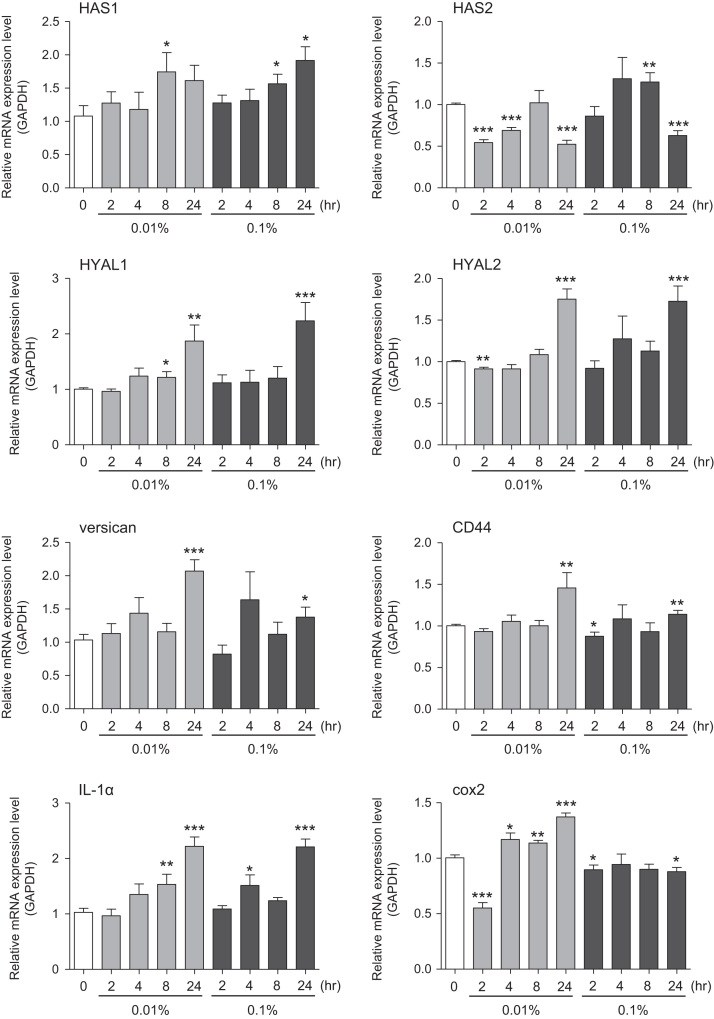

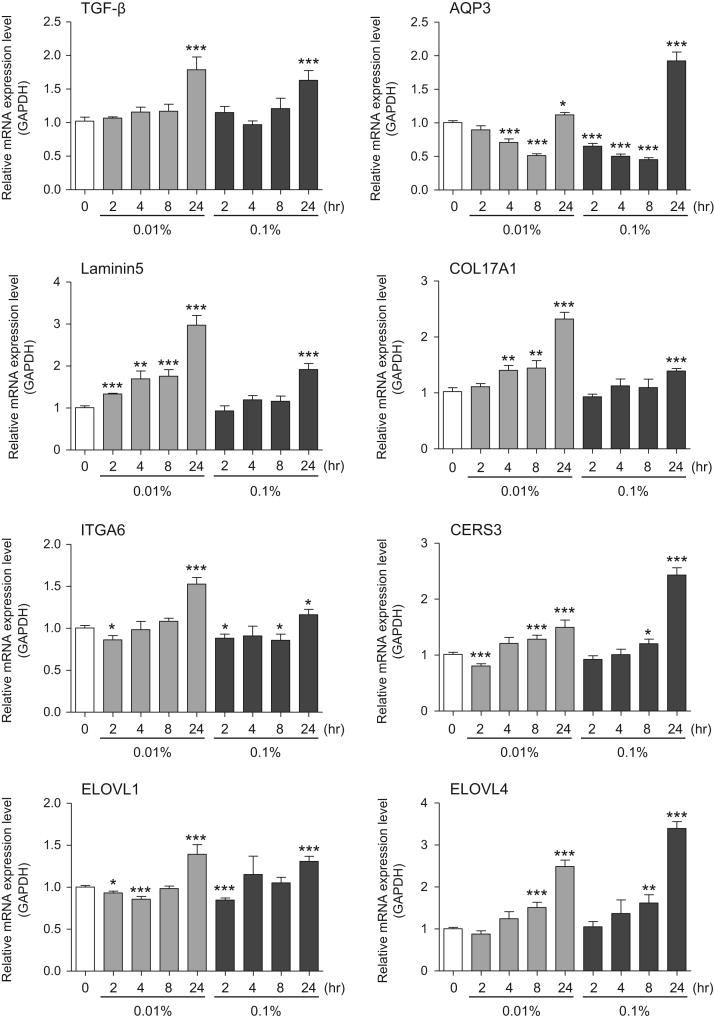

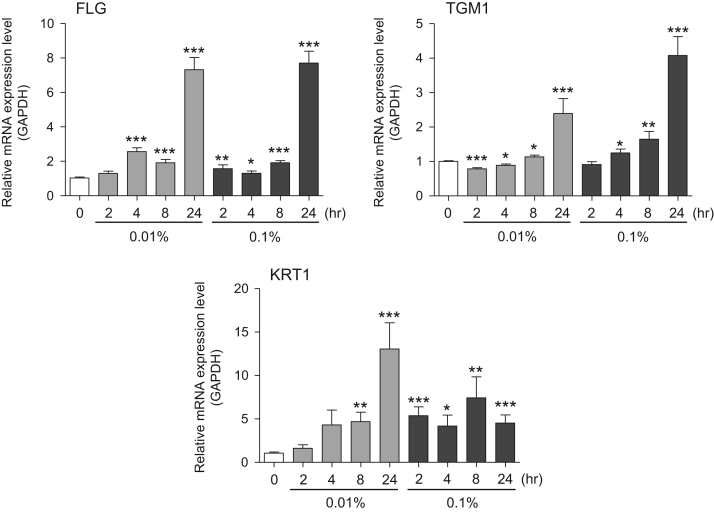
Gene expression levels in keratinocyte cells after exposure to malabar nut extract. The mRNA expression levels were normalized to GAPDH expression, and the relative gene expression levels in the cells at 2, 4, 8, and 24 h after initiation of extract exposure were compared to the corresponding levels for unexposed cells, whose levels were defined as 1.0.

**Fig. 5:**
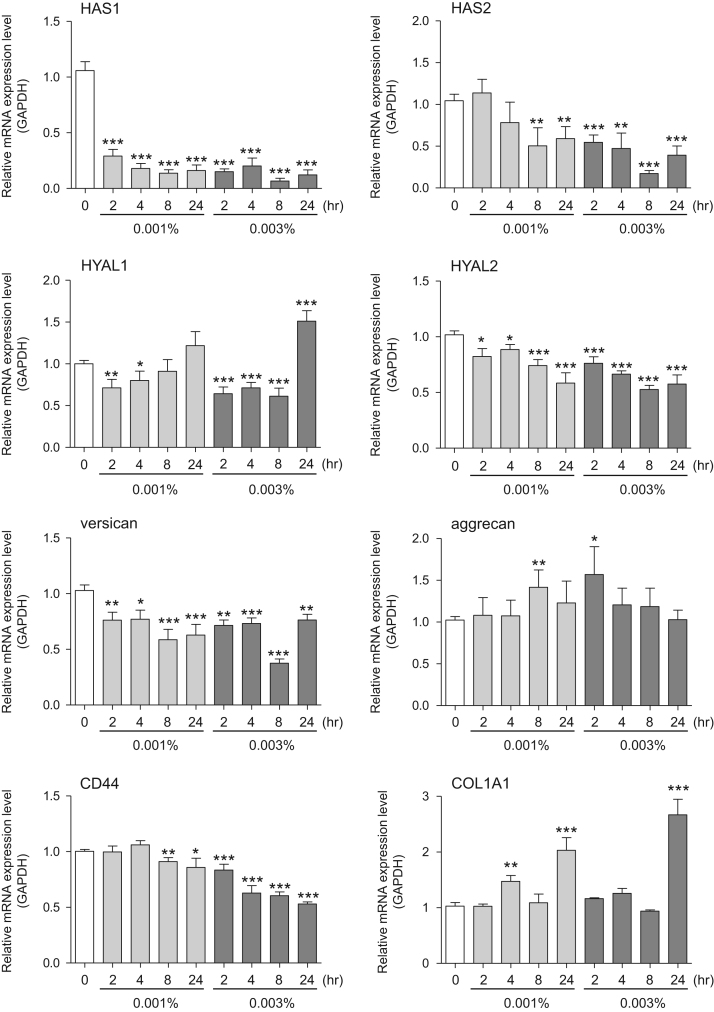

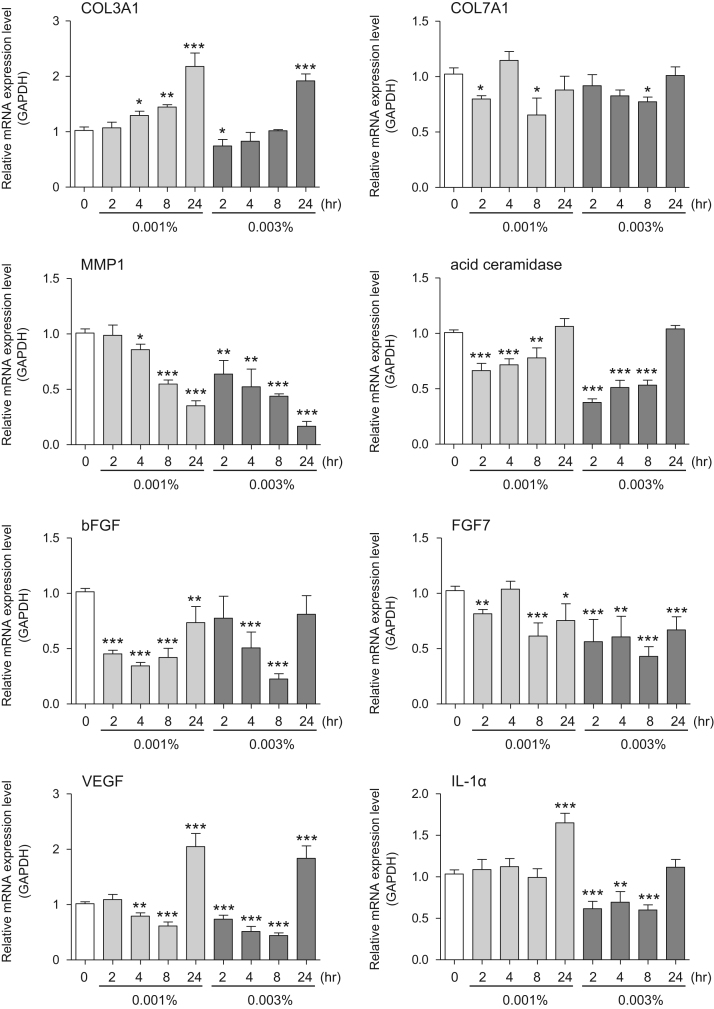

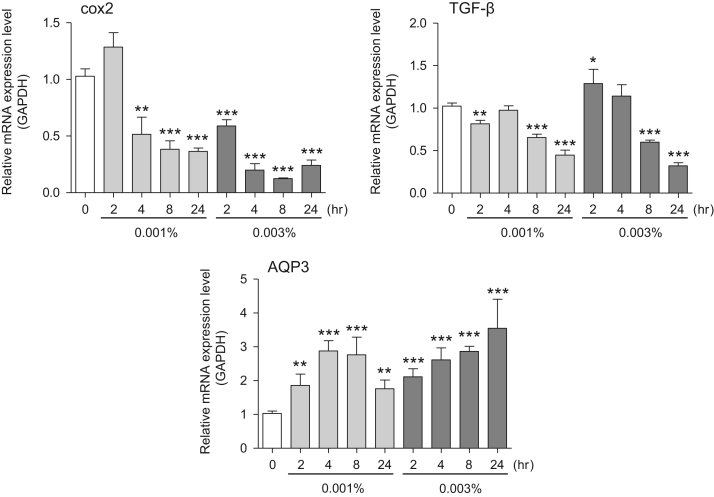
Gene expression levels in fibroblast cells after exposure to emblic myrobalan extract. The mRNA expression levels were normalized to GAPDH expression, and the relative gene expression levels in the cells at 2, 4, 8, and 24 h after initiation of extract exposure were compared to the corresponding levels for unexposed cells, whose levels were defined as 1.0.

**Fig. 6:**
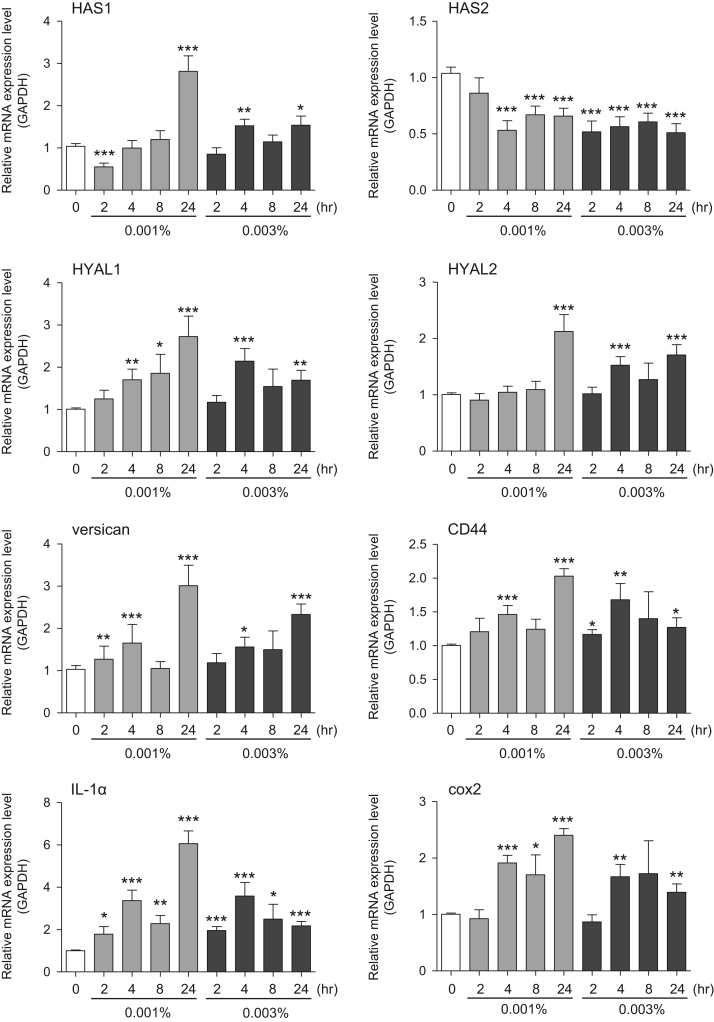

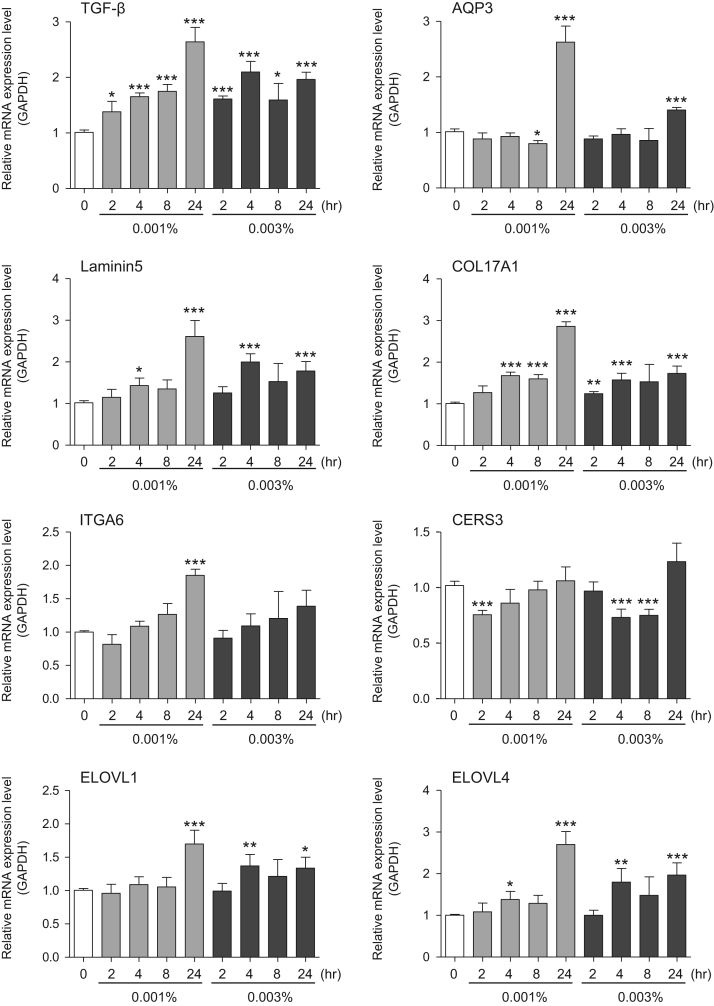

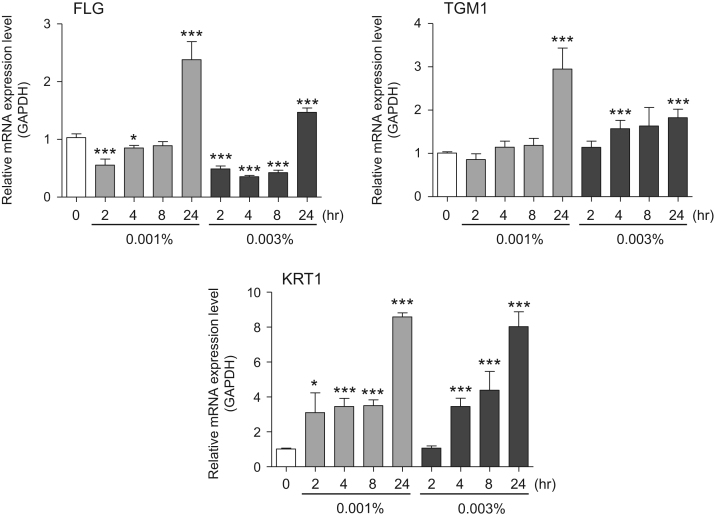
Gene expression levels in keratinocyte cells after exposure to emblic myrobalan extract. The mRNA expression levels were normalized to GAPDH expression, and the relative gene expression levels in the cells at 2, 4, 8, and 24 h after initiation of extract exposure were compared to the corresponding levels for unexposed cells, whose levels were defined as 1.0.

This data article contains bar graphs showing gene expression levels in fibroblasts and keratinocytes in response to exposure to 0.01% and 0.001% holy basil plant (*Ocimum tenuiflorum*) extract, 0.1% and 0.01% malabar nut plant (*Justicia adhatoda*) extract, and 0.003% and 0.001% emblic myrobalan plant (*Phyllanthus emblica*) extract, harvested in Negombo, Sri Lanka. For fibroblasts, the dataset includes expression profiles for genes encoding hyaluronan synthase 1 (HAS1), hyaluronan synthase 2 (HAS2), hyaluronidase-1 (HYAL1), hyaluronidase-2 (HYAL2), versican, aggrecan, CD44, collagen, type I, alpha 1 (COL1A1), collagen, type III, alpha 1 (COL3A1), collagen, type VII, alpha 1 (COL7A1), matrix metalloproteinase 1 (MMP1), acid ceramidase, basic fibroblast growth factor (bFGF), fibroblast growth factor-7 (FGF7), vascular endothelial growth factor (VEGF), interleukin-1 alpha (IL-1α), cyclooxygenase-2 (cox2), transforming growth factor beta (TGF-β), and aquaporin 3 (AQP3) (Fig. 1). For keratinocytes, the expression profiles are for genes encoding HAS1, HAS2, HYAL1, HYAL2, versican, CD44, IL-1α, cox2, TGF-β, AQP3, Laminin5, collagen, type XVII, alpha 1 (COL17A1), integrin alpha-6 (ITGA6), ceramide synthase 3 (CERS3), elongation of very long chain fatty acids protein 1 (ELOVL1), elongation of very long chain fatty acids protein 4 (ELOVL4), filaggrin (FLG), transglutaminase 1 (TGM1), and keratin 1 (KRT1) (Fig. 2). The data represent the mean ± SE values from triplicate independent experiments (*P < 0.05, **P < 0.001 and ***P < 0.001 vs. 0 time) (Fig. 3, Fig. 4, Fig. 5, Fig. 6).

## 2 Experimental design, materials and methods

### 2.1 Materials

Holy basil plants (*Ocimum tenuiflorum*) were harvested from a medicinal garden at the Institute of Traditional Plants in Sri Lanka (Negombo, Sri Lanka). The plant shoot metabolites were extracted by using 70% ethyl alcohol solution. Malabar nut plants (*Justicia adhatoda*) were harvested from a medicinal garden at the Institute of Traditional Plants in Sri Lanka (Negombo, Sri Lanka). The plant leave metabolites were extracted by using 70% ethyl alcohol solution. Emblic myrobalan plants (*Phyllanthus emblica*) were harvested from a medicinal garden at the Institute of Traditional Plants in Sri Lanka (Negombo, Sri Lanka). The plant leave metabolites were extracted by using 50% ethyl alcohol solution.

### 2.2 Fibroblast cell culture

Normal human skin fibroblasts, RIKEN original (NB1RGB), were provided by the RIKEN BRC through the National Bio-Resource Project of the MEXT, Japan. The cells were cultured in Minimum Essential Media-alpha (MEMα; Life Technologies Corp.) supplemented with 10% fetal bovine serum (FBS; Biowest) and 0.2% NaHCO3. The cells were grown at 37 °C in a humidified incubator containing 5% CO_2,_ according to the manufacturer's instructions. For all of the experiments, human fibroblasts were seeded into a 60 mm dish (5 × 10^4^ cells/dish) and incubated for 8 h with culture media containing 10% FBS. The cells were subsequently subjected to serum starvation for 16 h with serum-free MEMα.

### 2.3 Keratinocyte cell culture

Normal human epidermal keratinocytes (HEKn; GIBCO) were isolated from neonatal foreskin. The cells were cultured in Medium 154 (Invitrogen) supplemented with human keratinocyte growth factor (HKGS; Invitrogen), according to the manufacturer's instructions. The cells were grown at 37 °C in a humidified incubator containing 5% CO_2_. For all of the experiments, human keratinocytes were seeded into a collagen-coated 60 mm dish (5 × 10^4^ cells/dish), and incubated for 8 h with culture media containing HKGS. The cells were next subjected to HKGS starvation for 16 h with Medium 154.

### 2.4 Exposure of the cells to the plant extract, RNA isolation and quantitative real-time PCR

The cells were seeded into a 60 mm dish (5 × 10^4^ cells/dish). The cells were exposed to 0.01% or 0.001% of the plant extract, for 24 h at 37 °C. The cells were collected at 2, 4, 8, and 24 h after initiation of the exposure. Total RNA was extracted from the cells by using the TRI reagent (Merck). This RNA extract was used as a template for subsequent cDNA synthesis with oligo dT primers (Table 1), using the Primescript RT reagent Kit (Takara bio inc.). The mRNA levels were quantified using a LightCycler 96 system (Roche) and SYBR *Premix Ex Taq* II (Takara Bio Inc.). The data were analyzed using the delta cycle threshold method, and calculated based on the Cq values, and the expression of each gene was normalized to GAPDH. All values are reported as means ± standard error, as previously described [18].

**Table 1:** Nucleotide sequences of primers used in this study.

Primers	Sequences	Direction	Reference
***Quantitative real time-PCR***			
*HAS1*			
HAS1-F	3′-CGCTAACTACGTCCCTCTGC-5′	Sense	[1]
HAS1-R	3′-CCAGTACAGCGTCAACATGG-5′	Anti-sense	
*HAS2*			
HAS2-F	3′-GCCTCATCTGTGGAGATGGT-5′	Sense	[2]
HAS2-R	3′-ATGCACTGAACACACCCAAA-5′	Anti-sense	
*HYAL1*			
HYAL1-F	3′-CCAAGGAATCATGTCAGGCCATCAA-5′	Sense	[3]
HYAL1-R	3′-CCCACTGGTCACGTTCAGG-5′	Anti-sense	
*HYAL2*			
HYAL2-F	3′-GGCTTAGTGAGATGGACCTC-5′	Sense	[3]
HYAL2-R	3′-CCGTGTCAGGTAATCTTTGAG-5′	Anti-sense	
*Versican*			
VCAN 3-F	3′-TGAGAACCCTGTATCGTTTTGAGA-5′	Sense	[4]
VCAN 3-R	3′-CGTTAAGGCACGGGTTCATT-5′	Anti-sense	
*Aggrecan*			
ACAN-F	3′-TCGAGGACAGCGAGGCC-5′	Sense	[5]
ACAN-R	3′-TCGAGGGTGTAGCGTGTAGAGA-5′	Anti-sense	
*CD44*			
CD44-F	3′-GCTATTGAAAGCCTTGCAGAG-5′	Sense	[6]
CD44-R	3′-CGCAGATCGATTTGAATATAACC-5′	Anti-sense	
*COL1A1*			
COL1A1-F	3′-CACCAATCACCTGCGGTACAGAA-5′	Sense	[7]
COL1A1-R	3′-CAGATCACGTCATCGCACAAC-5′	Anti-sense	
*COL3A1*			
COL3A1-F	3′-CCCACTATTATTTTGGCACAACAG-5′	Sense	[8]
COL3A1-R	3′-AACGGATCCTGAGTCACAGACA-5′	Anti-sense	
*COL7A1*			
COL7A1-F	3′-CTCAGCAGCTATCACCTGGAC-5′	Sense	[9]
COL7A1-R	3′-TGTCCACCACACGTAGTTCAA-5′	Anti-sense	
*MMP1*			
MMP1-F	3′-TGTGGTGTCTCACAGCTTCC-5′	Sense	[3]
MMP1-R	3′-CTTGCCTCCCATCATTCTTC-5′	Anti-sense	
*Acid ceramidase*			
acid ceramidase-F	3′-CGTACAGAGGTGCAGTTCCA-5′	Sense	Original
acid ceramidase-R	3′-GTAGGCCAGGCAATTTTTCA-5′	Anti-sense	
*bFGF*			
bFGF-F	3′-AGAGCGACCCTCACATCAAG-5′	Sense	[10]
bFGF-R	3′-ACTGCCCAGTTCGTTTCAGT-5′	Anti-sense	
*FGF7*			
FGF7-F	3′-CATGAACACCCGGAGCACTAC-5′	Sense	[11]
FGF7-R	3′-CACTGTGTTCGACAGAAGAGTCTTC-5′	Anti-sense	
*VEGF*			
VEGF-F	3′-GGAGAGATGAGCTTCCTACAG-5′	Sense	[12]
VEGF-R	3′-TCACCGCCTTGGCTTGTCACA-5′	Anti-sense	
*IL-1α*			
IL-1α-F	3′-TGGCTCATTTTCCCTCAAAAGTTG-5′	Sense	[13]
IL-1α-R	3′-AGAAATCGTGAAATCCGAAGTCAAG-5′	Anti-sense	
*cox2*			
COX2-F	3′-TGAGCATCTACGGTTTGCTG-5′	Sense	[14]
COX2-R	3′-TGCTTGTCTGGAACAACTGC-5′	Anti-sense	
*TGF-β*			
TGF-*β*-F	3′-GCCCTGGACACCAACTATTG-5′	Sense	[15]
TGF-*β*-R	3′-GTCCAGGCTCCAAATGTAGG-5′	Anti-sense	
*AQP3*			
AQP3-F	3′-GTCACTCTGGGCATCCTCAT-5′	Sense	[16]
AQP3-R	3′-TATTCCAGCACCCAAGAAGG-5′	Anti-sense	
*Laminin5*			
Laminin5-F	3′-GCCTGGAGTACAACGAGGTC-5′	Sense	Original
Laminin5-R	3′-AGTTGGCAAACTTGATGAGGAC-5′	Anti-sense	
*COL17A1*			
COL17A1-F	3′-CGAGACTTTCGACTACTCAGAGC-5′	Sense	Original
COL17A1-R	3′-GAGGACGAGAACAAGCTGAC-5′	Anti-sense	
*ITGA6*			
ITGA6-F	3′-TCTCGCTGGGATCTTGATGC-5′	Sense	Original
ITGA6-R	3′-CCTAGAGCGTTTAAAGAATCCAC-5′	Anti-sense	
*CERS3*			
CERS3-F	3′-TCTCTGCTGACTGCATCTATTG-5′	Sense	Original
CERS3-R	3′-GAAGCCAGAATCTTTCCAACC-5′	Anti-sense	
*ELOVL1*			
ELOVL1-F	3′-GGACTTCTCTCTGGCCCTG-5′	Sense	Original
ELOVL1-R	3′-CGTGCTTCATCACCTCTTGG-5′	Anti-sense	
*ELOVL4*			
ELOVL4-F	3′-GATTCTCCCCCTGTTCACATC-5′	Sense	Original
ELOVL4-R	3′-TTCAGACCGAAGAATGAGTGAC-5′	Anti-sense	
*FLG*			
FLG-F	3′-GAAGGTGAAGGTCGGAGTC-5′	Sense	Original
FLG-R	3′-GAAGATGGTGATGGGATTTC-5′	Anti-sense	
*TGM1*			
TGM1-F	3′-CGAAGGCTCTGGGTTACAGA-5′	Sense	Original
TGM1-R	3′-TGTCACTGTTTCATTGCCTCC-5′	Anti-sense	
*KRT1*			
KRT1-F	3′-TGAGCTGAATCGTGTGATCC-5′	Sense	Original
KRT1-R	3′-CCAGGTCATTCAGCTTGTTC-5′	Anti-sense	
*GAPDH*			
GAPDH-F	3′-GAAGGTGAAGGTCGGAGTC-5′	Sense	[17]
GAPDH-R	3′- GAAGATGGTGATGGGATTTC-5′	Anti-sense	

### 2.5 Statistical analysis

All the values have been reported in terms of mean ± SE values. The data were analyzed using the Student's *t*-test. A *P* value less than 0.05 was considered to be statistically significant.
